# Power Muirhead mean in spherical normal fuzzy environment and its applications to multi-attribute decision-making: Spherical normal fuzzy power Muirhead mean

**DOI:** 10.1007/s40747-022-00688-8

**Published:** 2022-02-28

**Authors:** Tansu Temel, Salih Berkan Aydemir, Yaşar Hoşcan

**Affiliations:** 1 Department of computer Engineering, Eskisehir technical university, Eskisehir, Turkey; 2 Department of computer Engineering, Amasya university, Amasya, Turkey

**Keywords:** Multi-attribute decision-making, Normal distribution, Spherical Normal fuzzy set, Muirhead mean, Power aggregation

## Abstract

This study aims to propose the power Muirhead mean (PMM) operator in the spherical normal fuzzy sets (SNoFS) environment to solve multiple attribute decision-making problems. Spherical normal fuzzy sets better characterize real-world problems. On the other hand, the Muirhead mean (MM) considers the relationship between any number of criteria of the operator. Power aggregation (PA) reduces the negative impact of excessively high or excessively low values on aggregation results. This article proposes two new aggregation methods: spherical normal fuzzy power Muirhead mean (SNoFPMM) and spherical normal fuzzy weighted power Muirhead mean (SNoFWPMM). Also, these operators produce effective results in terms of their suitability to real-world problems and the relationship between their criteria. The proposed operators are applied to solve the problems in choosing the ideal mask for the COVID-19 outbreak and investment company selection. However, uncertainty about the effects of COVID-19 complicates the decision-making process. Spherical normal fuzzy sets can handle both real-world problems and situations involving uncertainty. Our approach has been compared with other methods in the literature. The superior aspects and applicability of our strategy are also mentioned.

## Introduction

The multi-attribute decision-making (MADM) mechanism is the process of finding the most suitable alternatives in complex scenarios by synthetically evaluating the values of multiple attributes of all alternatives [18]. In this decision-making process, subjective and biased attitudes cause uncertainty and inconsistency in the data. Therefore, fuzzy sets are used to handle uncertainty and vagueness data. The fuzzy set was presented by Lotfi Zadeh [66]. Atanassov proposed intuitionistic fuzzy sets (IFS) because the concept of fuzzy sets only consists of membership functions and covers a narrow set [6]. IFSs have the membership degree($\mu$) and the non-membership degree ($\nu$). In IFS, it is obtained depending on the membership and non-membership degree. IFSs are generalized as bivariate. Pythagorean fuzzy sets have a larger domain of $\mu$ and $\nu$ values [40]. Similarly, a larger space is represented by Fermatean fuzzy sets [8]. Ultimately, basic fuzzy sets are insufficient with values of $\mu =0.9$ and $\nu =0.8$. Hence, q-rung orthopair fuzzy sets (q-ROFSs) have been proposed by Yager [58]. However, the sets aforementioned contain a dependent hesitant degree. Because the membership variables are defined in a narrow space, and the hesitant degree is dependent, a fuzzy set with three variables is proposed. Fuzzy sets with an independent hesitant degree are called spherical fuzzy sets (SFSs) [24]. On the other hand, SFSs have been used with many aggregation operators. The interrelationship between any two criteria is considered with the Bonferroni Mean (BM) operator [16]. Harmonic Mean can be used to avoid outlier data [13]. *t*th order generalized spherical fuzzy sets are combined with the power MM operator [28]. The MM operator takes into account the relationship between the desired number of criteria. The interrelationship between criteria contains at most as many parameter vectors. Also, SFSs have been used with T-norm-based aggregation operators. Especially, Dombi [5], Einstein [39], Hamacher [45] T-norm can be given as instances. In another study, an Emergency decision support algorithm is proposed in the spherical fuzzy sets environment [4]. A detailed literature review regarding the methods presented in the article is examined in Table 1.

**Table 1:** Literature study for normal fuzzy sets

References	Approach	Application
[49]	Induced intuitionistic normal fuzzy	Supplier selection
[36]	Normal intuitionistic fuzzy Bonferroni mean	Stock investment
[67]	Normal intuitionistic fuzzy Heronian mean	Stock evaluation
[31]	Normal interval-valued intuitionistic fuzzy generalized aggregation	Supply company selection
[60]	Interval-valued Pythagorean normal fuzzy	Investment company selection
[62]	q-Rung orthopair normal fuzzy aggregation	Suitable global partner selection
[63]	q-Rung picture normal fuzzy Heronian mean	Healthcare product purchase
[30]	Normal neutrosophic Bonferroni mean	Investment company selection
[32]	Normal neutrosophic Heronian mean	Investment company selection
[34]	Normal neutrosophic frank aggregation	Investment company selection
[64]	Spherical normal fuzzy Bonferroni mean	COVID-19 mask selection

To summarize, normal fuzzy sets were combined by many methods. However, normal fuzzy sets are not addressed in power aggregation, Muirhead Mean, and spherical fuzzy environments. By adding standard deviation and variance values to fuzzy sets, decision matrices with normal distribution can be expected to produce more consistent results for real-world problems. Normal distribution was used with IFS [46], PFS [60], and q-ROFS [62]. As mentioned before, because IFSs and their general states contain a dependent hesitant degree, 3D spherical fuzzy sets give more consistent and reliable results for decision-makers in MADM problems. The normal distribution is applied to spherical fuzzy sets. In addition, the SNoFS and the BM operator are combined [64]. The aforementioned comprehensive literature summary shows that in the SNoFS environment, the power MM operator was not used to solve MADM. Aggregation operators and generalized fuzzy sets are frequently used in MCDM. However, the fact that there is a wide evaluation space that also addresses the hesitations in terms of decision-makers makes the evaluation of the problem more consistent and sensible. The assumption of this study is to provide consistent and valid solutions to real-life MCDM problems by adding the normal distribution to the combination of spherical fuzzy sets and a general aggregation operator structure (Muirhead mean). The motivation for the proposed method in the paper can be summarized.

1. The new MADM concept of spherical normal fuzzy sets with the MM operator is proposed.
2. With the spherical normal fuzzy sets, the degree of independent hesitation is handled and more consistent results are produced for the problems in daily life with the normal distribution.
3. Power aggregation reduces the negative effects of excessively high and excessively low criteria values and the MM operator examines the interrelationship between any number of criteria.
4. It has been applied to the issues of selecting the ideal mask for the COVID 19 pandemic and investment company selection.

## Preliminaries

This section gives the normal fuzzy number, spherical fuzzy sets, SNoFSs, and basic operations. Furthermore, power aggregation and MM are mentioned.

### Definition 1

[59] ${\mathbb {R}}$ is the set of real numbers. Let $Z=(\mu , \sigma )$ be a normal fuzzy number. The membership function of the normal fuzzy set can be defined as follows.

1 $$\begin{aligned} Z(x)=e^{-\left( \frac{x-\mu }{\sigma }\right) ^2} \end{aligned}$$

where $$x,\mu ,\sigma \in {\mathbb {R}}$$ and $$\sigma >0$$.

### Definition 2

[3] Let S be a finite space. Spherical fuzzy sets can be defined as

2 $$\begin{aligned} U=\{<x,s_u(x),i_u(x),d_u(x)>|x \in S\} \end{aligned}$$

where $s_u(x)$ is called positive, $i_u(x)$ is neutral, $d_u(x)$ is negative membership values. $s_u(x),i_u(x)$, $d_u(x) \in [0,1]$. $r_u(x)=\sqrt{1-(s_u^2(x)+i_u^2(x)+d_u^2(x)) }$ is its refusal degree.

### Spherical normal fuzzy number

#### Definition 3

[64] Let X be a finite set, $T=<(\mu _t,\sigma _t),(s_t,i_t, d_t)>$ is defined as the spherical normal fuzzy set. where $s_t(x)$ is called positive, $i_t(x)$ is neutral, $d_t(x)$ is negative membership values. Also,

3 $$\begin{aligned}&s_t(x)=s_te^{-\left( \frac{x-\mu }{\sigma }\right) ^2} \end{aligned}$$

4 $$\begin{aligned}&i_t(x)=1-(1-i_t)e^{-\left( \frac{x-\mu }{\sigma }\right) ^2} \end{aligned}$$

5 $$\begin{aligned}&d_t(x)=1-(1-d_t)e^{-\left( \frac{x-\mu }{\sigma }\right) ^2} \end{aligned}$$

#### Definition 4

[64] Let $S_1=<(\mu _1,\sigma _1),(s_1,i_1,d_1)>$ and $S_2=<(\mu _2,\sigma _2),(s_2,i_2,d_2)>$ be two SNoF numbers. The four basic operations on SNoFS are defined as follows $\lambda \ge 0$.

1. $$S_1 \oplus S_2=\left( (\mu _1+\mu _2,\sigma _1+\sigma _2),\sqrt{s^2_1+s^2_2-s^2_1s^2_2},i_1i_2, d_1d_2\right) $$
2. $$\begin{array}{l}S_1 \otimes S_2=\left( \left( \mu _1\mu _2,\mu _1\mu _2\sqrt{\frac{\sigma _1^2}{\mu _1^2}+\frac{\sigma _2^2}{\mu _2^2}}\right) ,\right. \\ \left. s_1s_2,\sqrt{i^2_1+i^2_2-i^2_1i^2_2}, \sqrt{d^2_1+d^2_2-d^2_1d^2_2}\right) \end{array}$$
3. $$\lambda S_1=\left( (\lambda \mu _1,\lambda \sigma _1),(\sqrt{1-(1-s_1^2)^\lambda },i_1^\lambda ,d_1^\lambda )\right) $$
4. $$S_1^\lambda =\left( (\mu _1^\lambda ,\lambda ^{\frac{1}{2}}\mu _1^{\lambda -1}\sigma _1), s_1^\lambda ,\sqrt{1-(1-i_1^2)^\lambda },\sqrt{1-(1-d_1^2)^\lambda }\right) $$

#### Definition 5

[64] Let $S_1=<(\mu ,\sigma ),(s,i,d)>$ be a SNoF. Score and accuracy functions for SNoF can be defined as follows. $Sc_1(S)=1+\mu (s^2-i^2-d^2)$, $Sc_2(S)=1+\sigma (s^2-i^2-d^2)$, $Acc_1(S)=1+\mu (s^2+i^2+d^2)$ and $Acc_2(S)=1+\sigma (s^2+i^2+d^2)$.

The two SNoF values can be compared with the score and accuracy functions mentioned above as follows.

#### Definition 6

[64] Let $S_1=<(\mu _1,\sigma _1),(s_1,i_1,d_1)>$ and $S_2=<(\mu _2,\sigma _2),(s_2,i_2,d_2)>$ be two SNoFS.

1. If $$Sc_1(S_1)>Sc_1(S_2)$$, then $$S_1>S_2$$.
2. If $Sc_1(S_1)=Sc_1(S_2)$ and $Acc_1(S_1)>Acc_1(S_2)$ then $S_1>S_2$.
3. If $Sc_1(S_1)=Sc_1(S_2)$ and $Acc_1(S_1)=Acc_1(S_2)$ then, If $Sc_2(S_1)<Sc_2(S_2)$, then $S_1>S_2$.
  If $Sc_2(S_1)=Sc_2(S_2)$ and $Acc_2(S_1)<Acc_2(S_2)$ then $S_1>S_2$.

### Muirhead mean operator

The MM operator was proposed by Muirhead for classical numbers [38]. The motivation of the MM operator is that considering the interrelationships of all arguments, it influences the aggregation result.

#### Definition 7

[38] Let $\alpha _i(i=1,2,\ldots ,n)$ be the set of nonnegative real numbers. $P=(p_1,p_2,\ldots ,p_n) \in {\mathbb {R}}^n$ be a vector of parameters. The MM operator is expressed as,

6 $$\begin{aligned} \mathrm{MM}^P(\alpha _1,\alpha _2,\ldots ,\alpha _n)=\left( \frac{1}{n!} \sum _{\phi \in S_n} \prod _{j=1}^{n} \alpha _{\phi (j)}^{P_j}\right) ^\frac{1}{\sum _{j=1}^{n}P_j} \end{aligned}$$

where $S_n$ is the set of all permutations. and $\phi (j)(j=1,2,\ldots ,n)$ is any permutation of $(1,2,\ldots ,n)$

The MM operator provides a general aggregation structure. Specific cases of the MM according to the P parameter can be expressed as follows.

1. If $P=(1,0,\ldots ,0)$, The MM operator is reduced to an arithmetic averaging operator.
  7 $$\begin{aligned}&\mathrm{MM}^{(1,0,\ldots ,0)}(\alpha _1,\alpha _2,\ldots ,\alpha _n)\nonumber \\&\quad =\frac{1}{n}\sum _{i=1}^{n}\alpha _i. \end{aligned}$$
2. If $P=(1/n,1/n,\ldots ,1/n)$, the MM operator is reduced to a geometric averaging operator.
  8 $$\begin{aligned} \mathrm{MM}^{(1/n,1/n,\ldots ,1/n)}(\alpha _1,\alpha _2,\ldots ,\alpha _n)=\frac{1}{n}\prod _{i=1}^{n}\alpha _i. \end{aligned}$$
3. If $P=(1,1,0,0,\ldots ,0)$, MM operator is reduced to Bonferroni mean operator [10].
  9 $$\begin{aligned}&\mathrm{MM}^{(1,1,0,0,\ldots ,0)}(\alpha _1,\alpha _2,\ldots ,\alpha _n)\nonumber \\&\quad =\left( \frac{1}{\alpha (\alpha +1)}\underset{x \ne y}{\sum _{x,y=1}^{n}}\alpha _x \alpha _y\right) ^{\frac{1}{2}} \end{aligned}$$
4. If $P=(\overbrace{1,1,\ldots ,1}^k,\overbrace{0,0,\ldots ,0}^{n-k})$, MM operator is reduced to Maclaurin symmetric mean operator [37].
  10 $$\begin{aligned}&\mathrm{MM}^{(\overbrace{1,1,\ldots ,1}^k,\overbrace{0,0,\ldots ,0}^{n-k})}(\alpha _1,\alpha _2,\ldots ,\alpha _n)\nonumber \\&\quad =\left( \sum _{1\le x_1<x_2<\cdots <x_k\le n}\prod _{y=1}^{k}\alpha _{x_y}\right) ^{\frac{1}{2}} \end{aligned}$$

### Dual Muirhead mean operator

The dual Muirhead mean DMM operator is the dual structure of the MM operator.

#### Definition 8

[38] Let $\alpha _i(i=1,2,\ldots ,n)$ be the set of nonnegative real numbers. $P=(p_1,p_2,\ldots ,p_n) \in {\mathbb {R}}^n$ be a vector of parameters.

11 $$\begin{aligned}&\mathrm{DMM}^P(\alpha _1,\alpha _2,\ldots ,\alpha _n)\nonumber \\&\quad =\frac{1}{\sum _{j=1}^{n}P_j}\left( \prod _{\phi \in S_n} \sum _{j=1}^{n} {P_j}\alpha _{\phi (j)}\right) ^{\frac{1}{n!}}, \end{aligned}$$

where $S_n$ is the set of all permutations. and $\phi (j)(j=1,2,\ldots ,n)$ is any permutation of $(1,2,\ldots ,n)$

### Power average operator

The power average operator was first proposed by Yager in 2001 [56]. The PA operator decreases the negative impact of outliers on aggregation results.

#### Definition 9

[56] Let $a_i(i=1,2,\ldots ,n)$ be a sets of real number ($a_i\ge 0$). PA operator is defined.

12 $$\begin{aligned} PA(a_1,a_2,\ldots ,a_n)=\frac{\sum _{i=1}^{n} a_i({1+T(a_i)})}{\sum _{i=1}^{n} (1+T(a_i))} \end{aligned}$$

Weighted power average (WPA) is defined as

13 $$\begin{aligned} \mathrm{WPA}(a_1,a_2,\ldots ,a_n)=\frac{\sum _{i=1}^{n} a_i({1+T(a_i)})w_i}{\sum _{i=1}^{n} (1+T(a_i))} \end{aligned}$$

where $w_i\in [0,1]$ is a weight vector $T(a_i)$.

$T(a_i)=\mathop {\sum _{j=1}^{m}\mathrm{Sup}(a_i, a_j)}_{i\ne j}$ and Sup$(a_i,a_j)$ is support measure that supplies the conditions: Sup$(a_i,a_j)\in [0,1];$ Sup$(a_i,a_j)=\mathrm{Sup}(a_j,a_i)$, $\mathrm{Sup}(a_i,a_j) \ge \mathrm{Sup}(a_k,a_l)$, if $\left| a_i-a_j\right| < \left| a_k-a_l\right|$.

$\mathrm{Sup}(a_i,a_j)=1-d(a_i,a_j)$, d is a distance formula. The distance between two spherical normal fuzzy sets can be defined.

#### Definition 10

Let $S_1=<(\mu _1,\sigma _1),(s_1,i_1,d_1)>$ and $S_2=<(\mu _2,\sigma _2),(s_2,i_2,d_2)>$ be two spherical normal fuzzy numbers. The normalized euclidean distance between two spherical normal fuzzy sets can be calculated as

14 $$\begin{aligned}&d(S_1,S_2)\nonumber \\&\quad =\frac{1}{4}\sqrt{[(dS_1)\mu _1-(dS_2)\mu _2]^2 + \frac{1}{2}((dS_1)\sigma _1-(dS_2)\sigma _2 )}\nonumber \\ \end{aligned}$$

where $$dS_1=(1+s_1^2-i_1^2-d_1^2)$$ and $$dS_2=(1+s_2^2-i_2^2-d_2^2)$$

## Novel spherical normal fuzzy power Muirhead mean operators

MM operators are frequently used in solving MADM problems. MM operators are used with generalized fuzzy sets such as intuitionistic fuzzy sets [29], Pythagorean fuzzy sets [25], q-rung orthopair fuzzy sets [48], picture fuzzy sets [52]. However, considering Table 1, the power MM operator in the normal spherical fuzzy sets environment has not been considered until now. In this section, weighted and unweighted PMM and PDMM operators are recommended in the spherical normal fuzzy environment.

### Definition 11

Let $S_k=<(\mu _k,\sigma _k),(s_k,i_k,d_k)> (k=1,2,\ldots ,n)$ be a set of SNoF numbers. $P=(p_1,p_2,\ldots ,p_n) \in {\mathbb {R}}^n$ be a vector of parameters. The spherical normal fuzzy power Muirhead Mean (SNoFPMM) operator is defined as

15 $$\begin{aligned}&\mathrm{SNoFPMM}(S_1,S_2,\ldots ,S_n)\nonumber \\&\quad =\left( \frac{1}{n!} \sum _{\phi \in S_n} \prod _{j=1}^{n} \left( n\delta _jS_{\phi (j)}\right) ^{P_j}\right) ^\frac{1}{\sum _{j=1}^{n}P_j} \end{aligned}$$

where $\delta _j=\frac{({1+T(S_j)})}{\sum _{t=1}^{n} (1+T(S_t))}$, $(\delta _1,\delta _2,\ldots ,\delta _n)^T$ is the power weight vector. $\phi (j)$ is any permutation of $(1,2,\ldots ,n)$

### Theorem 1

Let $S_k=<(\mu _k,\sigma _k),(s_k,i_k,d_k)> (k=1,2,\ldots ,n)$ be a set of SNoF numbers. $P=(p_1,p_2,\ldots ,p_n) \in {\mathbb {R}}^n$ be a vector of parameters. Then, the aggregated value by the SNoFPMM operator is still a SNoFN.

Hence,

16 $$\begin{aligned}&\mathrm{SNoFPMM}(S_1,S_2,\ldots ,S_n)=\Bigg \langle \left( \frac{1}{n!} \sum _{\phi \in S_n} \prod _{j=1}^{n} \left( n\delta _j\mu _{\phi (j)}\right) ^{P_j}\right) ^\frac{1}{\sum _{j=1}^{n}P_j}, \nonumber \\&\quad \sqrt{\frac{1}{\sum _{j=1}^{n}P_j}}\left( \left( \frac{1}{n!} \sum _{\phi \in S_n} \prod _{j=1}^{n} \left( n\delta _j\mu _{\phi (j)}\right) ^{P_j}\right) ^{\frac{1}{\sum _{j=1}^{n}P_j}-1}\right) \nonumber \\&\quad \cdot \left( \left( \frac{1}{n!}\sum _{\phi \in S_n} \prod _{j=1}^{n} \left( n\delta _j\mu _{\phi (j)}\right) ^{P_j}\right) \sum _{\phi \in S_n}\sqrt{\sum _{j=1}^{n}\frac{\sigma ^2_{\phi (j)} }{\mu ^2_{\phi (j)}}P_j}\right) ,\nonumber \\&\left( \sqrt{1-\left( \prod _{\sigma \in S_n} \left( 1-\prod _{j=1}^{n} \left( 1-\left( 1-s^2_{\phi (j)}\right) ^{n\delta _j} \right) ^{p_j}\right) \right) ^{1/n!}}\right) ^\frac{1}{\sum _{j=1}^{n}p_j},\nonumber \\&\sqrt{1-\left( 1-\prod _{\sigma \in S_n} \left( 1-\prod _{j=1}^{n} \left( 1-(i_{\phi (j)})^{2n\delta _j} \right) ^{p_j}\right) ^{1/n!}\right) ^\frac{1}{\sum _{j=1}^{n}p_j}},\nonumber \\&\sqrt{1-\left( 1-\prod _{\sigma \in S_n} \left( 1-\prod _{j=1}^{n} \left( 1-(d_{\phi (j)})^{2n\delta _j} \right) ^{p_j}\right) ^{1/n!}\right) ^\frac{1}{\sum _{j=1}^{n}p_j}} \Bigg \rangle \end{aligned}$$

### Proof

Considering operational laws for SNoFN, we have (From Definition 4)

$$\begin{aligned}&n\delta _jS_{\phi (j)}\\&\quad =\Bigg \langle (n\delta _j\mu _{\phi (j)},n\delta _j\sigma _{\phi (j)}),\\&\qquad \left( \left( \sqrt{1-\left( 1-s_{\phi (j)}^2\right) ^{n\delta _j}} \right) ,i_{\phi (j)}^{2n\delta _j},d_{\phi (j)}^{2n\delta _j}\right) \Bigg \rangle \end{aligned}$$

Also, (From Definition 4, operation 4)

$$\begin{aligned}&\left( n\delta _jS_{\phi (j)}\right) ^{P_j}\\&\quad =\Big \langle \left( \left( n\delta _j\mu _{\phi (j)}\right) ^{P_j},\sqrt{P_j} \left( n\delta _j\mu _{\phi (j)}\right) ^{P_j-1}.n\delta _j\sigma _{\phi (j)}\right) ,\\&\qquad \left( \left( \sqrt{1-\left( 1-s_{\phi (j)}^2\right) ^{n\delta _j}} \right) ^{P_j},\sqrt{1-\left( 1-i_{\phi (j)}^{2n\delta _j}\right) ^{P_j}},\right. \\&\qquad \left. \sqrt{1-\left( 1-s_{\phi (j)}^{2n\delta _j}\right) ^{P_j}}\right) \Big \rangle \end{aligned}$$

Therefore, (From Definition 4, operation 2)

$$\begin{aligned}&\prod _{j=1}^{n}\left( n\delta _jS_{\phi (j)}\right) ^{P_j}\\&\quad =\Bigg \langle \left( \prod _{j=1}^{n}\left( n\delta _j\mu _{\phi (j)}\right) ^{P_j},\prod _{j=1}^{n}\left( n\delta _j\mu _{\phi (j)}\right) ^{P_j}.\sqrt{\sum _{j=1}^{n}\frac{\sigma _{\phi (j)}^2}{\mu _{\phi (j)}^2}P_j}\right) ,\\&\qquad \left( \prod _{j=1}^{n}\left( \sqrt{1-\left( 1-s_{\phi (j)}^2\right) ^{n\delta _j}} \right) ^{P_j},\sqrt{1-\prod _{j=1}^{n}\left( 1-i_{\phi (j)}^{2n\delta _j}\right) ^{P_j}},\right. \\&\qquad \left. \sqrt{1-\prod _{j=1}^{n}\left( 1-s_{\phi (j)}^{2n\delta _j}\right) ^{P_j}}\right) \Bigg \rangle \end{aligned}$$

and In Definition 4, operations 3 and 4 are applied.

Finally,

$$\begin{aligned}&\frac{1}{n!}\left( \sum _{\phi \in S_n}\prod _{j=1}^{n}\left( n\delta _jS_{\phi (j)}\right) ^{P_j}\right) ^\frac{1}{\sum _{j=1}^{n}P_j}\\&\quad =\Bigg \langle \left( \frac{1}{n!} \sum _{\phi \in S_n} \prod _{j=1}^{n} \left( n\delta _j\mu _{\phi (j)}\right) ^{P_j}\right) ^\frac{1}{\sum _{j=1}^{n}P_j},\\&\qquad \sqrt{\frac{1}{\sum _{j=1}^{n}P_j}}\left( \left( \frac{1}{n!} \sum _{\phi \in S_n} \prod _{j=1}^{n} \left( n\delta _j\mu _{\phi (j)}\right) ^{P_j}\right) ^{\frac{1}{\sum _{j=1}^{n}P_j}-1}\right) .\\&\qquad \left( \left( \frac{1}{n!}\sum _{\phi \in S_n} \prod _{j=1}^{n} \left( n\delta _j\mu _{\phi (j)}\right) ^{P_j}\right) \sum _{\phi \in S_n}\sqrt{\sum _{j=1}^{n}\frac{\sigma ^2_{\phi (j)} }{\mu ^2_{\phi (j)}}P_j}\right) ,\\&\qquad \left( \sqrt{1-\left( \prod _{\sigma \in S_n} \left( 1-\prod _{j=1}^{n} \left( 1-\left( 1-s^2_{\phi (j)}\right) ^{n\delta _j} \right) ^{p_j}\right) \right) ^{1/n!}}\right) ^\frac{1}{\sum _{j=1}^{n}p_j},\\&\qquad \sqrt{1-\left( 1-\prod _{\sigma \in S_n} \left( 1-\prod _{j=1}^{n} \left( 1-(i_{\phi (j)})^{2n\delta _j} \right) ^{p_j}\right) ^{1/n!}\right) ^\frac{1}{\sum _{j=1}^{n}p_j}},\\&\qquad \sqrt{1-\left( 1-\prod _{\sigma \in S_n} \left( 1-\prod _{j=1}^{n} \left( 1-(d_{\phi (j)})^{2n\delta _j} \right) ^{p_j}\right) ^{1/n!}\right) ^\frac{1}{\sum _{j=1}^{n}p_j}} \Bigg \rangle \end{aligned}$$

$$\square $$

With the help of Definition 4, Theorem 1 is provided. In Theorem 1, the sum and product symbols mentioned in the $\left( \frac{1}{n!}\sum _{\phi \in S_n} \prod _{j=1}^{n} \left( n\delta _jS_{\phi (j)}\right) ^{P_j}\right) ^\frac{1}{\sum _{j=1}^{n}P_j}$ Muirhead Mean structures are represented by $\oplus$ and $\otimes$, respectively.

### Theorem 2

Let $S_k=<(\mu _k,\sigma _k),(s_k,i_k,d_k)> (k=1,2,...n)$ be a set of SNoF numbers. $P=(p_1,p_2,\ldots ,p_n) \in {\mathbb {R}}^n$ be a vector of parameters. Let $W=(w_1,w_2,\ldots ,w_n)$ be the weight of the criteria. The spherical normal fuzzy weighted power Muirhead mean (SNoFWPMM) operator is defined as

17 $$\begin{aligned} \begin{aligned}&\mathrm{SNoFWPMM}(S_1,S_2,\ldots ,S_n)\\&\quad =\left( \frac{1}{n!} \sum _{\phi \in S_n} \prod _{j=1}^{n} \left( n\delta ^W_jS_{\phi (j)}\right) ^{P_j}\right) ^\frac{1}{\sum _{j=1}^{n}P_j} \end{aligned} \end{aligned}$$

where $\delta ^W_j=\frac{W_j({1+T(S_j)})}{\sum _{t=1}^{n} W_t(1+T(S_t))}$, $(\delta _1,\delta _2,...\delta _n)^T$ is the power weight vector. $\phi (j)$ is any permutation of $(1,2,\ldots ,n)$ and $\sum _{j=1}^{n}W_j=1$.

### Proof

The proof of this theorem is proved similarly to Theorem 1. $\square$

### Definition 12

Let $S_k=<(\mu _k,\sigma _k),(s_k,i_k,d_k)> (k=1,2,...n)$ be a set of SNoF numbers. $P=(p_1,p_2,\ldots ,p_n) \in {\mathbb {R}}^n$ be a vector of parameters. The spherical normal fuzzy power dual Muirhead mean (SNoFPDMM) operator is defined as

18 $$\begin{aligned}&\mathrm{SNoFPDMM}(S_1,S_2,\ldots ,S_n)\nonumber \\&\quad =\frac{1}{\sum _{j=1}^{n}P_j}\left( \prod _{\phi \in S_n} \sum _{j=1}^{n} \left( {P_j}S^{n\delta _j}_{\phi (j)}\right) \right) ^\frac{1}{n!} \end{aligned}$$

where $\delta _j=\frac{({1+T(S_j)})}{\sum _{t=1}^{n} (1+T(S_t))}$, $(\delta _1,\delta _2,...\delta _n)^T$ is the power weight vector. $\phi (j)$ is any permutation of $(1,2,\ldots ,n)$

### Theorem 3

Let $S_k=<(\mu _k,\sigma _k),(s_k,i_k,d_k)> (k=1,2,...n)$ be a set of SNoF numbers. $P=(p_1,p_2,\ldots ,p_n) \in {\mathbb {R}}^n$ be a vector of parameters. Then, the aggregated value by the SNoFPDMM operator is still a SNoFN.

Hence,

$$\begin{aligned}&\mathrm{SNoFPDMM}(S_1,S_2,\ldots ,S_n)\\&\quad = \Bigg \langle \left( \frac{1}{n!} \sum _{\phi \in S_n} \prod _{j=1}^{n} \left( n\delta _j\mu _{\phi (j)}\right) ^{P_j}\right) ^\frac{1}{\sum _{j=1}^{n}P_j},\\&\qquad \sqrt{\frac{1}{\sum _{j=1}^{n}P_j}}\left( \left( \frac{1}{n!} \sum _{\phi \in S_n} \prod _{j=1}^{n} \left( n\delta _j\mu _{\phi (j)}\right) ^{P_j}\right) ^{\frac{1}{\sum _{j=1}^{n}P_j}-1}\right) \\&\qquad . \left( \left( \frac{1}{n!}\sum _{\phi \in S_n} \prod _{j=1}^{n} \left( n\delta _j\mu _{\phi (j)}\right) ^{P_j}\right) \sum _{\phi \in S_n}\sqrt{\sum _{j=1}^{n}\frac{\sigma ^2_{\phi (j)} }{\mu ^2_{\phi (j)}}P_j}\right) ,\\&\qquad \sqrt{1-\left( 1-\prod _{\sigma \in S_n} \left( 1-\prod _{j=1}^{n} \left( 1-(s_{\phi (j)})^{2n\delta _j} \right) ^{p_j}\right) ^{1/n!}\right) ^\frac{1}{\sum _{j=1}^{n}p_j}},\\&\qquad \left( \sqrt{1-\left( \prod _{\sigma \in S_n} \left( 1-\prod _{j=1}^{n} \left( 1-\left( 1-i^2_{\phi (j)}\right) ^{n\delta _j} \right) ^{p_j}\right) \right) ^{1/n!}}\right) ^\frac{1}{\sum _{j=1}^{n}p_j},\\&\qquad \left( \sqrt{1-\left( \prod _{\sigma \in S_n} \left( 1-\prod _{j=1}^{n} \left( 1-\left( 1-d^2_{\phi (j)}\right) ^{n\delta _j} \right) ^{p_j}\right) \right) ^{1/n!}}\right) ^\frac{1}{\sum _{j=1}^{n}p_j} \Bigg \rangle \end{aligned}$$

### Proof

The proof of this theorem is proved similarly to Theorem 1. $\square$

### Theorem 4

Let $S_k=<(\mu _k,\sigma _k),(s_k,i_k,d_k)> (k=1,2,...n)$ be a set of SNoF numbers. $P=(p_1,p_2,\ldots ,p_n) \in {\mathbb {R}}^n$ be a vector of parameters. Let $W=(w_1,w_2,\ldots ,w_n)$ be the weight of the criteria. The spherical normal fuzzy weighted Dual power Muirhead Mean (SNoFWDPMM) operator is defined as

19 $$\begin{aligned} \begin{aligned}&\mathrm{SNoFWDPMM}(S_1,S_2,\ldots ,S_n) \\&\quad =\frac{1}{\sum _{j=1}^{n}P_j}\left( \prod _{\phi \in S_n} \sum _{j=1}^{n} \left( {P_j}S_{\phi (j)}\right) ^{n\delta ^W_j}\right) ^\frac{1}{n!} \end{aligned} \end{aligned}$$

where $\delta ^W_j=\frac{W_j({1+T(S_j)})}{\sum _{t=1}^{n} W_t(1+T(S_t))}$, $(\delta _1,\delta _2,...\delta _n)^T$ is the power weight vector. $\phi (j)$ is any permutation of $(1,2,\ldots ,n)$ and $\sum _{j=1}^{n}W_j=1$.

### Proof

The proof of this theorem is proved similarly to Theorem 1. $\square$

It can be easily demonstrated that the proposed operators provide the boundedness and idempotency properties. However, a monotonicity feature is not provided. Thus, the decision-making steps for the SNoFPMM, SNoFDPMM, SNoFWPMM and SNoFWDPMM operators can be explained.

**Fig. 1:**
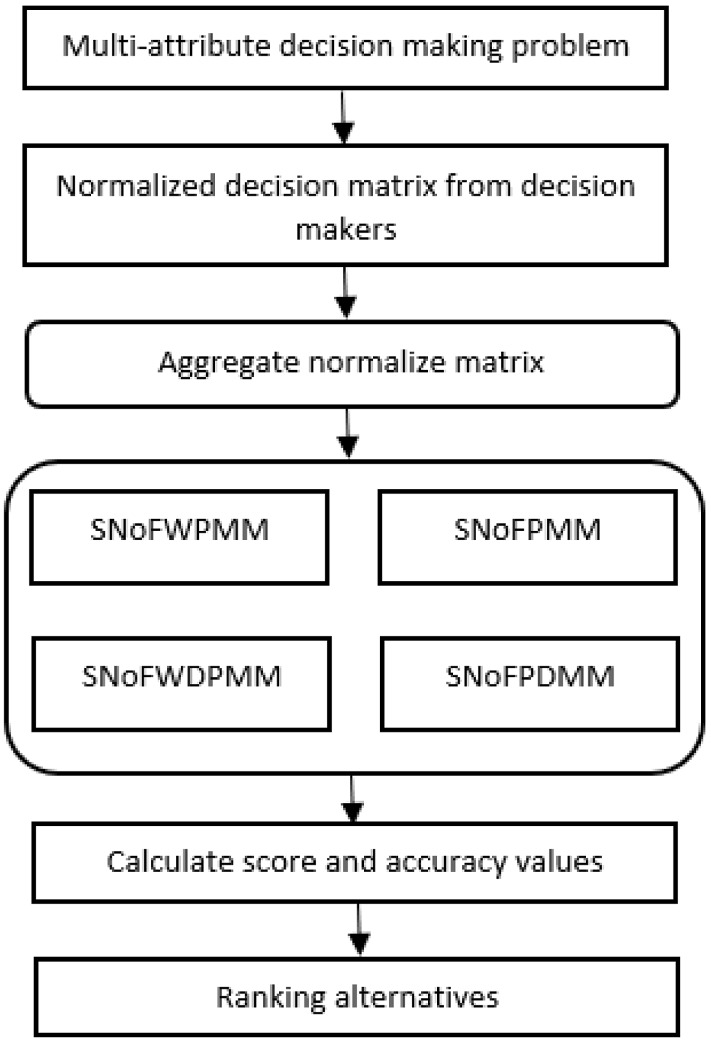
Flow-chart of the proposed algorithm

## A novel MADM hybrid approach based on the SNoFPMM and SNoFWPMM operators

MADM mechanism based on proposed aggregation operators is explained step by step. Firstly, let’s consider a classic MADM problem in the SNoF environment. Suppose the set of alternatives is $A=\{A_1,A_2,\ldots ,A_m\}$ and the set of criteria is $C=\{C_1,C_2,\ldots ,C_n\}$. $W_j$ is represented as the weights of the criteria. therefore, the evaluation given to the criterion $C_j$ for the $A_i$ alternative is represented by $\psi _{ij}=<(\mu _{ij},\sigma _{ij}),(s_{ij},i_{ij},d_{ij})>$. The decision matrix can be expressed as20Secondly, we examined the steps of the decision-making mechanism for the proposed operators.

*Step 1.* In the first step, different types of data are normalized. Then, a normalized operation is performed on the decision matrix as benefit type ($J_1$) and cost type ($J_2$)[51].

21 $$\begin{aligned} {\hat{\psi }}_{ij}= {\left\{ \begin{array}{ll} \Bigg \langle \frac{\mu _{ij}}{\underset{i}{\max }(\mu _{ij})},\frac{\sigma _{ij}}{\underset{i}{\max }(\sigma _{ij})}\frac{\sigma _{ij}}{\mu _{ij}} \Bigg \rangle \, (s_{ij},i_{ij},d_{ij});&{} \mathrm{for} \, J_1 \, \mathrm{attribute} \\ \Bigg \langle \frac{\underset{i}{\min }(\mu _{ij})}{\mu _{ij}},\frac{\sigma _{ij}}{\underset{i}{\max }(\sigma _{ij})}\frac{\sigma _{ij}}{\mu _{ij}} \Bigg \rangle \, (d_{ij},i_{ij},s_{ij});&{} \mathrm{for} \, J_2 \, \mathrm{attribute} \end{array}\right. }\nonumber \\ \end{aligned}$$

*Step 2.* Calculate the supports

22 $$\begin{aligned} \mathrm{Sup}(\psi _{ij},\psi _{ik})=1-d(\psi _{ij},\psi _{ik}),\quad j,k=1,2,...n. \end{aligned}$$

*Step 3.* Determine $\psi _{ij}$ of SNoFPMM number by other SNoFPMM numbers $\psi _{ij}^t$.

23 $$\begin{aligned} T(\psi _{ij})=\sum _{k=1,k\ne j}^{n}\mathrm{Sup}(\psi _{ij}, \psi _{ik}) \end{aligned}$$

*Step 4.* Utilize weights are determined with both weights and without weights.

24 $$\begin{aligned} \delta _{ij}=\frac{W_j(1+T(\psi _{ij}))}{\sum _{j=1}^{n}W_j(1+T(\psi _{ij}))} \end{aligned}$$

or

25 $$\begin{aligned} \delta _{ij}=\frac{(1+T(\psi _{ij}))}{\sum _{j=1}^{n}(1+T(\psi _{ij}))} \end{aligned}$$

where $$\sum _{j=1}^{n}w_{j}=1$$.

*Step 5.* Aggregation results are computed based on Eqs. 15, 17, 18, or 19 operators.

*Step 6.* According to Definitions 5 and 6, The score of alternatives is calculated.

*Step 7.* The best alternative is determined according to the rank results.

The steps of the proposed aggregation operators are given in Fig. 1. Four types of aggregation operators are used, weighted and without weighted.

**Fig. 2:**
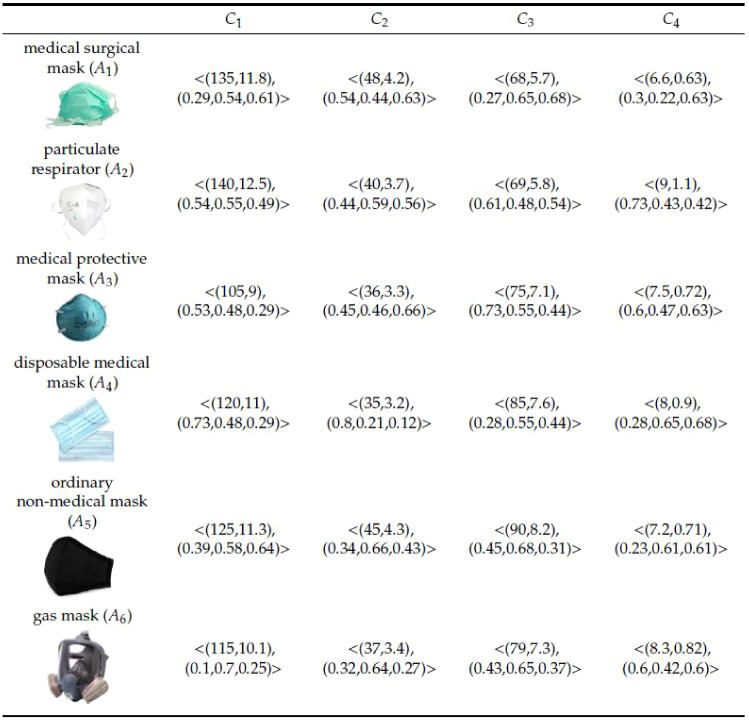
Spherical normal fuzzy decision matrix, (from [64])

## Application of proposed operators in ideal COVID-19 mask selection problem

The SNoFPMM and SNoFWPMM aggregation operators are used to choose the ideal mask in the COVID 19 outbreak. The COVID 19 outbreak first appeared in Wuhan, China, in December 2019. More than 190 countries worldwide are affected by this infectious disease [44]. It seems that the use of masks to reduce the spread of COVID 19 has a very important effect [2, 15, 26]. Using the same standard mask as healthcare professionals may not be very useful in daily life. On the other hand, factors such as whether the mask is filtered, usefulness, and raw material quality are also effective. Especially in this area, there are many studies on the usage of masks [9, 17]. Six types of masks can be considered under different criteria and purposes for COVID 19 epidemic [9, 14, 47, 64].

Commonly compared mask types are selected as medical-surgical masks, particulate respirators, medical protective masks, disposable medical masks, ordinary non-medical masks, and gas masks [64]. The particulate respirator is FFP2, FFP3, N95, N99 masks [54]. The evaluation of the related masks was based on four criteria. These criteria are leakage rate $(C_1)$, reusability $(C_2)$, raw material quality $(C_3)$, filtration efficiency $(C_4)$. Some criteria can be stated more clearly. The leakage rate means that the mask is designed to cover the human face. The filtration efficiency of non-oily $0.3\mu m$ particles is more than $95\%$. In selecting the ideal mask, the weight vector is determined by decision-makers, $w=(0.25,0.2,0.3,0.25)$. A decision matrix based on criteria and alternatives is given in Fig. 2 [64]. The decision matrix contains both Gaussian distribution and triple values of the spherical fuzzy set. SNoFPMM and SNoFWPMM operators are used in decision-making mechanisms. The MADM procedure is given step by step below.

*Step 1.* In the first step, different types of data are normalized. Normalized operation is performed on the decision matrix as benefit type ($J_1$) and cost type ($J_2$) [51]. The normalized decision matrix is given in Table 2. The first and third columns of the decision matrix are in the first column of the normalized decision matrix. The second and fourth columns of the decision matrix are in the second column of the normalized decision matrix.

**Table 2:** Normalized decision matrix

$$T=<(\mu _t,\sigma _t),(s_t,i_t,d_t)>$$	
$(A_1,C_1)<(0.964,0.083), (0.29,0.54,0.61)>$	$(A_1,C_2)<(1, 0.085), (0.54,0.44,0.63)>$
$$(A_1,C_3)<(0.756, 0.058),(0.27,0.65,0.68)>$$	$$(A_1,C_4)<(0.733,0.05), (0.3,0.22,0.63)> $$
$(A_2,C_1)<(1, 0.089), (0.54,0.55,0.49)>$	$(A_2,C_2)<(0.833, 0.08), (0.44,0.59,0.56)>$
$$(A_2,C_3)<(0.767, 0.059),(0.61,0.48,0.54)>$$	$$(A_2,C_4)<(1,0.12), (0.73,0.43,0.42)> $$
$(A_3,C_1)<(0.75, 0.062), (0.53,0.48,0.29)>$	$(A_3,C_2)<(0.75, 0.07), (0.45,0.46,0.66)>$
$$(A_3,C_3)<(0.838, 0.082),(0.73,0.55,0.44)>$$	$$(A_3,C_4)<(0.833,0.06), (0.6,0.47,0.63)> $$
$(A_4,C_1)<(0.857, 0.081),(0.73,0.48,0.29)>$	$(A_4,C_2)<(0.729, 0.068),(0.8,0.21,0.12)>$
$$(A_4,C_3)<(0.944, 0.083),(0.28,0.55,0.44)>$$	$$(A_4,C_4)<(0.889, 0.09), (0.28,0.65,0.68)> $$
$(A_5,C_1)<(0.893, 0.082),(0.39,0.58,0.64)>$	$(A_5,C_2)<(0.938, 0.096),(0.34,0.66,0.43)>$
$$(A_5,C_3)<(1, 0.091), (0.45,0.68,0.31)>$$	$$(A_5,C_4)<(0.8,0.06), (0.23,0.61,0.61)> $$
$(A_6,C_1)<(0.821, 0.071),(0.1,0.7,0.25)>$	$(A_6,C_2)<(0.771, 0.073),(0.32,0.64,0.27)>$
$$(A_6,C_3)<(0.878, 0.082),(0.43,0.65,0.37)>$$	$$(A_6,C_4)<(0.922,0.07), (0.6,0.42,0.6)> $$

26 $$\begin{aligned} {\hat{\psi }}_{ij}= {\left\{ \begin{array}{ll} \Bigg \langle \frac{\mu _{ij}}{\underset{i}{\max }(\mu _{ij})},\frac{\sigma _{ij}}{\underset{i}{\max }(\sigma _{ij})}\frac{\sigma _{ij}}{\mu _{ij}}\Bigg \rangle \,(s_{ij},i_{ij},d_{ij});&{} \mathrm{for} \, J_1 \, \mathrm{attribute} \\ \Bigg \langle \frac{\underset{i}{\min }(\mu _{ij})}{\mu _{ij}},\frac{\sigma _{ij}}{\underset{i}{\max }(\sigma _{ij})}\frac{\sigma _{ij}}{\mu _{ij}} \Bigg \rangle \, (d_{ij},i_{ij},s_{ij});&{} \mathrm{for} \, J_2 \, \mathrm{attribute} \end{array}\right. }\nonumber \\ \end{aligned}$$

*Step 2.* Calculate the supports. Support values are given in below.

27 $$\begin{aligned}&\mathrm{Sup}(\psi _{ij},\psi _{ik})=1-d(\psi _{ij},\psi _{ik}),\, j,k=1,2,...n.\nonumber \\&\mathrm{Sup}(\psi _{11},\psi _{12})=\mathrm{Sup}(\psi _{12},\psi _{11})= 0.9315\nonumber \\&\mathrm{Sup}(\psi _{11},\psi _{13})=\mathrm{Sup}(\psi _{13},\psi _{11})= 0.9287 \nonumber \\&\mathrm{Sup}(\psi _{11},\psi _{14})=\mathrm{Sup}(\psi _{14},\psi _{11})= 0.9809\nonumber \\&\mathrm{Sup}(\psi _{12},\psi _{13})=\mathrm{Sup}(\psi _{13},\psi _{12})= 0.8549 \nonumber \\&\mathrm{Sup}(\psi _{12},\psi _{14})=\mathrm{Sup}(\psi _{14},\psi _{12})= 0.9358\nonumber \\&\mathrm{Sup}(\psi _{13},\psi _{14})=\mathrm{Sup}(\psi _{14},\psi _{13})= 0.9215 \nonumber \\&\mathrm{Sup}(\psi _{21},\psi _{22})=\mathrm{Sup}(\psi _{22},\psi _{21})= 0.9187\nonumber \\&\mathrm{Sup}(\psi _{21},\psi _{23})=\mathrm{Sup}(\psi _{23},\psi _{21})= 0.9668 \nonumber \\&\mathrm{Sup}(\psi _{21},\psi _{24})=\mathrm{Sup}(\psi _{24},\psi _{21})= 0.9059\nonumber \\&\mathrm{Sup}(\psi _{22},\psi _{23})=\mathrm{Sup}(\psi _{23},\psi _{22})= 0.9501 \nonumber \\&\mathrm{Sup}(\psi _{22},\psi _{24})=\mathrm{Sup}(\psi _{24},\psi _{22})= 0.8265\nonumber \\&\mathrm{Sup}(\psi _{23},\psi _{24})=\mathrm{Sup}(\psi _{24},\psi _{23})= 0.8815 \nonumber \\&\mathrm{Sup}(\psi _{31},\psi _{32})=\mathrm{Sup}(\psi _{32},\psi _{31})= 0.9188\nonumber \\&\mathrm{Sup}(\psi _{31},\psi _{33})=\mathrm{Sup}(\psi _{33},\psi _{31})= 0.9774 \nonumber \\&\mathrm{Sup}(\psi _{31},\psi _{34})=\mathrm{Sup}(\psi _{34},\psi _{31})= 0.9655\nonumber \\&\mathrm{Sup}(\psi _{32},\psi _{33})=\mathrm{Sup}(\psi _{33},\psi _{32})= 0.8935 \nonumber \\&\mathrm{Sup}(\psi _{32},\psi _{34})=\mathrm{Sup}(\psi _{34},\psi _{32})= 0.9513\nonumber \\&\mathrm{Sup}(\psi _{33},\psi _{34})=\mathrm{Sup}(\psi _{34},\psi _{33})= 0.9280 \nonumber \\&\mathrm{Sup}(\psi _{41},\psi _{42})=\mathrm{Sup}(\psi _{42},\psi _{41})= 0.9785\nonumber \\&\mathrm{Sup}(\psi _{41},\psi _{43})=\mathrm{Sup}(\psi _{43},\psi _{41})= 0.8702 \nonumber \\&\mathrm{Sup}(\psi _{41},\psi _{44})=\mathrm{Sup}(\psi _{44},\psi _{41})= 0.7762\nonumber \\&\mathrm{Sup}(\psi _{42},\psi _{43})=\mathrm{Sup}(\psi _{43},\psi _{42})= 0.8432 \nonumber \\&\mathrm{Sup}(\psi _{42},\psi _{44})=\mathrm{Sup}(\psi _{44},\psi _{42})= 0.7491\nonumber \\&\mathrm{Sup}(\psi _{43},\psi _{44})=\mathrm{Sup}(\psi _{44},\psi _{43})= 0.9006 \nonumber \\&\mathrm{Sup}(\psi _{51},\psi _{52})=\mathrm{Sup}(\psi _{52},\psi _{51})= 0.9858\nonumber \\&\mathrm{Sup}(\psi _{51},\psi _{53})=\mathrm{Sup}(\psi _{53},\psi _{51})= 0.9355 \nonumber \\&\mathrm{Sup}(\psi _{51},\psi _{54})=\mathrm{Sup}(\psi _{54},\psi _{51})= 0.9640\nonumber \\&\mathrm{Sup}(\psi _{52},\psi _{53})=\mathrm{Sup}(\psi _{53},\psi _{52})= 0.9591 \nonumber \\&\mathrm{Sup}(\psi _{52},\psi _{54})=\mathrm{Sup}(\psi _{54},\psi _{52})= 0.9379\nonumber \\&\mathrm{Sup}(\psi _{53},\psi _{54})=\mathrm{Sup}(\psi _{54},\psi _{53})= 0.8946 \nonumber \\&\mathrm{Sup}(\psi _{61},\psi _{62})=\mathrm{Sup}(\psi _{62},\psi _{61})= 0.9840\nonumber \\&\mathrm{Sup}(\psi _{61},\psi _{63})=\mathrm{Sup}(\psi _{63},\psi _{61})= 0.9640 \nonumber \\&\mathrm{Sup}(\psi _{61},\psi _{64})=\mathrm{Sup}(\psi _{64},\psi _{61})= 0.9083\nonumber \\&\mathrm{Sup}(\psi _{62},\psi _{63})=\mathrm{Sup}(\psi _{63},\psi _{62})= 0.9887 \nonumber \\&\mathrm{Sup}(\psi _{62},\psi _{64})=\mathrm{Sup}(\psi _{64},\psi _{62})= 0.9325\nonumber \\&\mathrm{Sup}(\psi _{63},\psi _{64})=\mathrm{Sup}(\psi _{64},\psi _{63})= 0.9494 \end{aligned}$$

*Step 3.* Compute the support $\psi _{ij}$ of SNoFPMM number by other SNoFPMM numbers $\psi _{ij}^t$.

28 $$\begin{aligned}&T(\psi _{ij})=\sum _{k=1,k\ne j}^{n}\mathrm{Sup}(\psi _{ij}, \psi _{ik})\nonumber \\&T(\psi _{ij})=\begin{bmatrix} 2.8411 &{}\quad 2.7117 &{}\quad 2.7275 &{}\quad 2.8502\\ 2.7914 &{}\quad 2.7052 &{}\quad 2.8185 &{}\quad 2.5532\\ 2.8616 &{}\quad 2.7721 &{}\quad 2.7630 &{}\quad 2.8811\\ 2.6249 &{}\quad 2.5604 &{}\quad 2.6390 &{}\quad 2.4593\\ 2.8853 &{}\quad 2.8640 &{}\quad 2.7703 &{}\quad 2.8426\\ 2.8563 &{}\quad 2.8887 &{}\quad 2.8772 &{}\quad 2.7726\\ \end{bmatrix} \end{aligned}$$

*Step 4.* Utilize weights are calculated with both weights and without weights. Utilize weights are given in Table 3.

**Table 3:** Weighted and non-weighted results for utilize weights

Non-weighted			
$\delta _{11}=0.2539$	$\delta _{12}=0.2435$	$\delta _{13}=0.2464$	$\delta _{14}=0.2545$
$\delta _{21}=0.2550$	$\delta _{22}=0.2492$	$\delta _{23}=0.2568$	$\delta _{24}=0.2390$
$\delta _{31}=0.2528$	$\delta _{32}=0.2469$	$\delta _{33}=0.2463$	$\delta _{34}=0.2540$
$\delta _{41}=0.2538$	$\delta _{42}=0.2493$	$\delta _{43}=0.2548$	$\delta _{44}=0.2422$
$\delta _{51}=0.2529$	$\delta _{52}=0.2515$	$\delta _{53}=0.2454$	$\delta _{54}=0.2501$
$\delta _{61}=0.2505$	$\delta _{62}=0.2526$	$\delta _{63}=0.2519$	$\delta _{64}=0.2451$

Weighted			
$\delta _{11}=0.2538$	$\delta _{12}=0.1962$	$\delta _{13}=0.2956$	$\delta _{14}=0.2544$
$\delta _{21}=0.2546$	$\delta _{22}=0.1991$	$\delta _{23}=0.3077$	$\delta _{24}=0.2986$
$\delta _{31}=0.2528$	$\delta _{32}=0.1975$	$\delta _{33}=0.2956$	$\delta _{34}=0.2541$
$\delta _{41}=0.2535$	$\delta _{42}=0.1992$	$\delta _{43}=0.3054$	$\delta _{44}=0.2419$
$\delta _{51}=0.2532$	$\delta _{52}=0.2015$	$\delta _{53}=0.2949$	$\delta _{54}=0.2504$
$\delta _{61}=0.2505$	$\delta _{62}=0.2021$	$\delta _{63}=0.3023$	$\delta _{64}=0.2451$

**Table 4:** Aggregation results

Non-weighted	Weighted
$<(0.8549, 0.0678),(0.3309,0.5106,0.6536)>$	$<(0.8459, 0.0671),(0.3350,0.5007,0.6423)>$
$<(0.8937, 0.0865),(0.5557,0.5515,0.5343)>$	$<(0.8833, 0.0855),(0.5658,0.5298,0.5149)>$
$<(0.7915, 0.0689),(0.5515,0.5105,0.5763)>$	$<(0.7836, 0.0682),(0.5627,0.4952,0.5541)>$
$<(0.8508, 0.0803),(0.4615,0.5092,0.4531)>$	$<(0.8412, 0.0794),(0.4643,0.5048,0.4503)>$
$<(0.904, 0.0819),(0.3351,0.6506,0.5540)>$	$<(0.8966, 0.0812),(0.3403,0.6391,0.5344)>$
$<(0.8461, 0.0744),(0.2949,0.6369,0.4279)>$	$<(0.8375, 0.0736),(0.2997,0.6241,0.4122)>$

*Step 5.* Aggregation results are calculated based on Eqs. 14 or 16 operators. Aggregation results are given in Table 4.

*Step 6.* According to Definitions 5 and 6, scores are calculated

*Step 7.* The best alternative is determined according to the rank results.

Non-weighted: $$A_3=0.8135 \succ A_2=0.7983 \succ A_4=0.7941 \succ A_6=0.6027 \succ A_1=0.5289 \succ A_5=0.4769$$.

Weighted: $$A_4=0.7884 \succ A_3=0.7739 \succ A_2=0.7519 \succ A_6=0.5798 \succ A_1=0.5107 \succ A_5=0.4460$$.

The step-by-step decision-making process with SNoFPMM and SNoFWPMM operators for the vector $R=(1,1,1,1)$ is examined. It can be seen that in the SNoFWPMM method, the ideal mask is a disposable medical mask.

On the other hand, it can be seen that the ideal mask with the SNoFPMM method is a medical protective mask. Also, considering Tables 6 and 7, the parameter analysis result is generally the ideal mask disposable medical mask. However, when the weight vector is not used, the ideal mask is a medical protective mask if all criteria are affected by each other (Parameter = (1,1,1,1)). The choice of a mask to be used in the COVID 19 epidemic reveals a real-world problem. When choosing the ideal mask with the suggested operators, the interaction between the criteria is essential. The Muirhead Mean operator provides this interaction with the parameter vector. Both parameter vector selection and criteria weights have an effect on the final ranking. If the decision-makers include the weight vector, the weight effect of the 3rd criterion is expected to be more than the other criteria. However, due to the use of the power aggregation operator with the Muirhead Mean, only the biased weights of the decision-maker are not considered, thanks to the Utilized weights.

The closeness between the score values depends on the relationship between the criteria. For example, in Table 6, as the number of criteria affected by each other increases, the difference between the score values decreases. On the contrary, in dual structures, as the number of criteria related to each other increases, the difference between the score values of the alternatives increases. However, dual structures have a similar ranking as non-dual structures. Generally speaking, the ideal mask was chosen as an ordinary non-medical mask.

**Table 5:** Characteristic comparison with basic methods

Methods	Spherical FN	Normal FN	Whether captures interrelationship of two attributes	Whether captures interrelationship of multiple attributes	Capability to reduce the negative effect
PFS [57]	✗	✗	✗	✗	✗
Picture FS [11]	✗	✗	✗	✗	✗
INFN with entropy [50]	✗	✓	✗	✗	✗
INFN [61]	✗	✓	✗	✗	✗
INFN and HM [67]	✗	✓	✓	✗	✗
IFP [55]	✗	✗	✗	✗	✓
T-SFPMM [28]	✓	✗	✓	✓	✓
SpNoF BM [64]	✓	✓	✓	✗	✗
SNoFPMM and SNoFWPMM	✓	✓	✓	✓	✓

**Table 6:** The impact of the R parameter on the SPFPMM results (COVID data)

Parameter	SNoFPMM		SNoWFPMM	
	Score	Rank	Score	Rank
(1, 0, 0, 0)	$S(A_1)=0.5935$	$A_4 \succ A_3 \succ A_2 \succ A_6 \succ A_1 \succ A_5$	$S(A_1)=0.5800$	$A_4 \succ A_3 \succ A_2 \succ A_6 \succ A_1 \succ A_5$
	$S(A_2)=0.8758$		$S(A_2)=0.8858$	
	$S(A_3)=0.9278$		$S(A_3)=0.9428$	
	$S(A_4)=1.0282$		$S(A_4)=0.9759$	
	$S(A_5)=0.5603$		$S(A_5)=0.5694$	
	$S(A_6)=0.7460$		$S(A_6)=0.7429$	
(1, 1, 0, 0)	$S(A_1)=0.5447$	$A_4 \succ A_3 \succ A_2 \succ A_6 \succ A_1 \succ A_5$	$S(A_1)=0.5295$	$A_4 \succ A_3 \succ A_2 \succ A_6 \succ A_1 \succ A_5$
	$S(A_2)=0.8373$		$S(A_2)=0.8216$	
	$S(A_3)=0.8636$		$S(A_3)=0.8521$	
	$S(A_4)=0.9201$		$S(A_4)=0.8957$	
	$S(A_5)=0.5057$		$S(A_5)=0.4807$	
	$S(A_6)=0.6839$		$S(A_6)=0.6691$	
(1, 1, 1, 0)	$S(A_1)=0.5343$	$A_4 \succ A_3 \succ A_2 \succ A_6\succ A_1 \succ A_5$	$S(A_1)=0.5178$	$A_4 \succ A_3 \succ A_2 \succ A_6\succ A_1 \succ A_5$
	$S(A_2)=0.8161$		$S(A_2)=0.7842$	
	$S(A_3)=0.8331$		$S(A_3)=0.8049$	
	$S(A_4)=0.8451$		$S(A_4)=0.8345$	
	$S(A_5)=0.4887$		$S(A_5)=0.4598$	
	$S(A_6)=0.6450$		$S(A_6)=0.6208$	
(1, 1, 1, 1)	$S(A_1)=0.5289$	$A_3 \succ A_2 \succ A_4 \succ A_6 \succ A_1 \succ A_5$	$S(A_1)=0.5107$	$A_4 \succ A_3 \succ A_2 \succ A_6 \succ A_1 \succ A_5$
	$S(A_2)=0.7983$		$S(A_2)=0.7519$	
	$S(A_3)=0.8135$		$S(A_3)=0.7739$	
	$S(A_4)=0.7941$		$S(A_4)=0.7884$	
	$S(A_5)=0.4769$		$S(A_5)=0.4460$	
	$S(A_6)=0.6027$		$S(A_6)=0.5798$

**Table 7:** The impact of the R parameter on the SNoFPDMM results (COVID data)

Parameter	SNoFPDMM		SNoFWDPMM	
	Score	Rank	Score	Rank
(1, 0, 0, 0)	$S(A_1)=0.5091$	$A_3 \succ A_2 \succ A_4 \succ A_6 \succ A_1 \succ A_5$	$S(A_1)=0.4966$	$A_3 \succ A_2 \succ A_4 \succ A_6 \succ A_5 \succ A_1$
	$S(A_2)=0.8325$		$S(A_2)=0.8466$	
	$S(A_3)=0.8519$		$S(A_3)=0.8694$	
	$S(A_4)=0.7493$		$S(A_4)=0.7170$	
	$S(A_5)=0.4929$		$S(A_5)=0.4980$	
	$S(A_6)=0.6077$		$S(A_6)=0.6058$	
(1, 1, 0, 0)	$S(A_1)=0.5649$	$A_4 \succ A_3 \succ A_2 \succ A_6 \succ A_1 \succ A_5$	$S(A_1)=0.5826$	$A_3 \succ A_4 \succ A_2 \succ A_6 \succ A_1 \succ A_5$
	$S(A_2)=0.8514$		$S(A_2)=0.8731$	
	$S(A_3)=0.8765$		$S(A_3)=0.8938$	
	$S(A_4)=0.8978$		$S(A_4)=0.8898$	
	$S(A_5)=0.5268$		$S(A_5)=0.5458$	
	$S(A_6)=0.6928$		$S(A_6)=0.7028$	
(1, 1, 1, 0)	$S(A_1)=0.6028$	$A_4 \succ A_3 \succ A_2 \succ A_6\succ A_1 \succ A_5$	$S(A_1)=0.6359$	$A_4 \succ A_3 \succ A_2 \succ A_6\succ A_1 \succ A_5$
	$S(A_2)=0.8633$		$S(A_2)=0.8875$	
	$S(A_3)=0.8930$		$S(A_3)=0.9094$	
	$S(A_4)=1.0087$		$S(A_4)=1.0211$	
	$S(A_5)=0.5449$		$S(A_5)=0.5712$	
	$S(A_6)=0.7275$		$S(A_6)=0.7489$	
(1, 1, 1, 1)	$S(A_1)=0.6395$	$A_4 \succ A_3 \succ A_2 \succ A_6 \succ A_1 \succ A_5$	$S(A_1)=0.6844$	$A_4 \succ A_3 \succ A_2 \succ A_6 \succ A_1 \succ A_5$
	$S(A_2)=0.8739$		$S(A_2)=0.8994$	
	$S(A_3)=0.9104$		$S(A_3)=0.9270$	
	$S(A_4)=1.1023$		$S(A_4)=1.1351$	
	$S(A_5)=0.5587$		$S(A_5)=0.5897$	
	$S(A_6)=0.7548$		$S(A_6)=0.7800$

**Table 8:** Rank comparison with other methods

Methods	Ranking
Archimedean norm based aggregation [3]	$A_4 \succ A_6 \succ A_3 \succ A_2 \succ A_1 \succ A_5$
Logarithmic function based aggregation [22]	$A_4 \succ A_3 \succ A_2 \succ A_6 \succ A_1 \succ A_5$
Cosine similarity based measures [41]	$A_4 \succ A_2 \succ A_1 \succ A_3 \succ A_5 \succ A_6$
Induced generalized based aggregation [51]	$A_3 \succ A_1 \succ A_4 \succ A_2 \succ A_5 \succ A_6$
Bonferroni Mean based aggregation [64]	$A_4 \succ A_3 \succ A_6 \succ A_2 \succ A_1 \succ A_5$
SNoFPMM aggregation (1, 0, 0, 0)	$A_4 \succ A_3 \succ A_2 \succ A_6 \succ A_1 \succ A_5$
SNoFWPMM aggregation (1, 0, 0, 0)	$A_4 \succ A_3 \succ A_2 \succ A_6 \succ A_1 \succ A_5$

## Comparative analysis with other methods

The proposed aggregation operators are compared with some methods studied in the literature. Firstly, the SNoFPMM and SNoFWPMM aggregation operators are compared with basic fuzzy sets handling uncertain and incomplete data. Then, the spherical and normal fuzzy sets studies are compared numerically. Table 5 contains basic fuzzy sets that deal with uncertainty. Pythagorean fuzzy set (PFS) is the total of the squares of membership and non-membership degrees. The picture fuzzy set handles uncertain data more consistent with having an independent hesitant degree. intuitionistic normal fuzzy numbers (INFNs) are a more reasonable approach for real-life data. On the other hand, the BM operator considers the interrelationship between any two criteria. The MM takes account of the interrelation between any number of arguments. However, considering the studies in Table 5, the proposed SNoFPMM operator includes all the operators mentioned above and fuzzy sets. SNoFPMM and SNoFWPMM operators both affect aggregation results with power Muirhead Mean and the spherical normal fuzzy set is more compatible for real-life problems.

The SNoFPMM and SNoFWPMM operators are compared with the normal fuzzy environment and interaction-based aggregation operators in Table 8. Archimedean t-norm /t-conorm, logarithmic operators, and cosine similarity-based measures were used in [3, 22, 41] study. However, their proposed approaches are considered only in the spherical fuzzy environment. In the [51] study, normal fuzzy sets are handled, but induced aggregation operators are used in an intuitionistic fuzzy set environment. Since the hesitant degree is dependent on IFS, it offers a narrow evaluation space for examining incomplete data. The operator proposed in the [64] study was examined in both spherical and normal fuzzy environments. However, the BM operator only examines the interrelationship between the two criteria. In the method we propose, the interrelationship between any number of criteria is taken into account, and the negative effects of extremely high or low values are reduced.

**Fig. 3:**
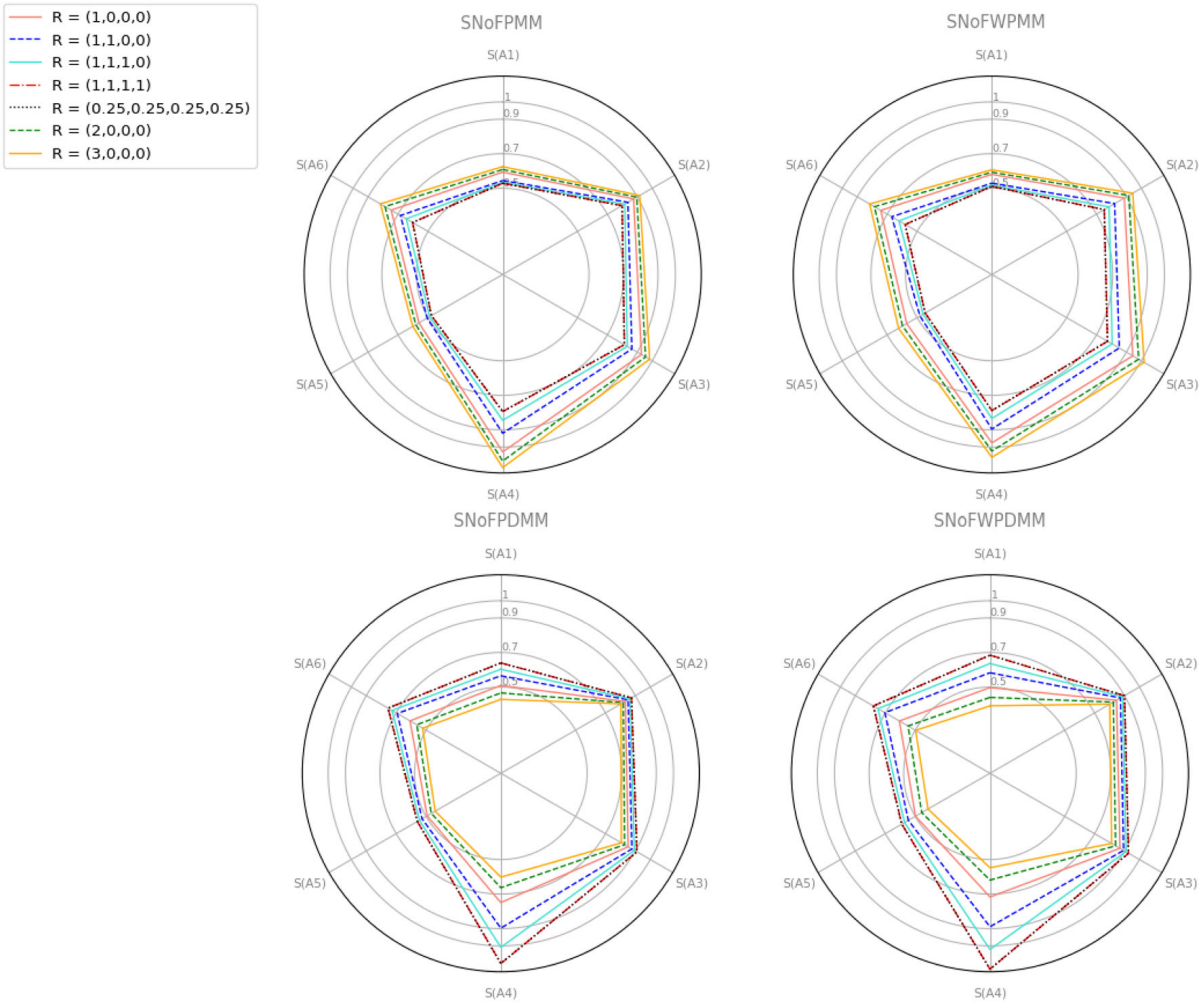
Graphical representation-effect of the R parameter for COVID data

## The effect of the R parameter on aggregation result

In Fig. 3, the SNoFPMM operator is represented according to the parameter vector R. In the vector $R=(r_1,0,0,0), r_1>0$ the interrelationship between the criteria is not taken into account. The vector $R=(1,1,0,0)$ examines the interrelationship between any two criteria. Similarly, $R=(1,1,1,0)$ examines the interrelationship between any three vectors and $R=(1,1,1,1)$ examines the interrelationship between all vectors. Considering all vector values, it is seen that the best mask is a disposable medical mask. It can also be seen that the $R=(0.25,0.25,0.25,0.25)$ and $R=(1,1,1,1)$ vectors have the same score values. Thanks to the MM operator, all criteria are handled in the same formulation. The second mask is the medical protective mask. The problem of mask selection has been addressed by considering the choice of the general public. Medical masks are generally recommended for those over 60 age or healthcare professionals [54]. Respirators are designed to protect healthcare professionals who care for COVID-19 patients in their environment [54].

## Application of investment company selection

We have added a second example to verify the compatibility and effectiveness of the proposed aggregation operators with the real world. The investment company selection problem has always been the subject of research by scholars. Jun Yei addresses this problem using correlation coefficients in normal neutrosophic sets [65]. Sahin proposed the generalized prioritized aggregation operator in the normal neutrosophic cluster environment [43]. On the other hand, Liu and Teng put forward a generalized power averaging operator in normal neutrosophic sets [33]. Liu and Li some normal proposed neutrosophic Bonferroni mean operators [33]. Let’s explore which industry a company should invest in. Our aim is to choose the ideal area to invest in. An MCDM decision matrix with four companies and three criteria is created. Alternatives are (1) $A_1$ is a car company, (2) $A_2$ is a food company, (3) $A_3$ is a computer company, and (4)$A_4$ is an arms company. There are three evaluation criteria. (1) $C_1$ is the risk, (2) $C_2$ is the growth, (3) $C_3$ is the environment. $C_1$ and $C_2$ are benefit criteria, $C_3$ is cost criterion. The weights of the criteria are determined as $w=(0.35,0.25,0.4)$. The decision matrix is given in Table 9. The decision matrix, which is evaluated as a neutrosophic set in other studies, is used as a spherical normal fuzzy set by changing the 4th row, 3rd column in this study. We changed the falsity-membership value from 0.8 to 0.7.

*Step 1.* Considering Eq. 26, the normalized decision matrix is obtained as in Table 10.

*Step 2.* Calculated supports:

$$\begin{aligned} \begin{array}{l} \mathrm{Sup}(\psi _{11},\psi _{12})=\mathrm{Sup}(\psi _{12},\psi _{11})= 0.9095 \\ \mathrm{Sup}(\psi _{11},\psi _{13})=\mathrm{Sup}(\psi _{13},\psi _{11})= 0.9484 \\ \mathrm{Sup}(\psi _{12},\psi _{13})=\mathrm{Sup}(\psi _{13},\psi _{12})= 0.8890 \\ \mathrm{Sup}(\psi _{21},\psi _{22})=\mathrm{Sup}(\psi _{22},\psi _{21})= 0.9388 \\ \mathrm{Sup}(\psi _{21},\psi _{23})=\mathrm{Sup}(\psi _{23},\psi _{21})= 0.9401 \\ \mathrm{Sup}(\psi _{22},\psi _{23})=\mathrm{Sup}(\psi _{23},\psi _{22})= 0.9397 \\ \mathrm{Sup}(\psi _{31},\psi _{32})=\mathrm{Sup}(\psi _{32},\psi _{31})= 0.9460 \\ \mathrm{Sup}(\psi _{31},\psi _{33})=\mathrm{Sup}(\psi _{33},\psi _{31})= 0.9166 \\ \mathrm{Sup}(\psi _{32},\psi _{33})=\mathrm{Sup}(\psi _{33},\psi _{32})= 0.9780 \\ \mathrm{Sup}(\psi _{41},\psi _{42})=\mathrm{Sup}(\psi _{42},\psi _{41})= 0.9195 \\ \mathrm{Sup}(\psi _{41},\psi _{43})=\mathrm{Sup}(\psi _{43},\psi _{41})= 0.8908 \\ \mathrm{Sup}(\psi _{42},\psi _{43})=\mathrm{Sup}(\psi _{43},\psi _{42})= 0.9682 \\ \end{array} \end{aligned}$$

*Step 3.* Calculate supports *T*: $T(\psi _{ij})$ = $$\begin{bmatrix} 1.8579 &{} 1.8030 &{} 1.8616 \\ 1.8579 &{} 1.8702 &{} 1.8328 \\ 1.8626 &{} 1.9533 &{} 1.8140 \\ 1.8103 &{} 1.9186 &{} 1.8757 \\ \end{bmatrix}$$

*Step 4.* Utilize weights are calculated. Non-weighted and weighted,respectively

$$\begin{aligned} \begin{array}{l@{\quad }l@{\quad }l@{\quad }|l@{\quad }l@{\quad }l} \delta _{11}=0.3353 &{} \delta _{12}=0.3289 &{} \delta _{13}=0.3358 &{} \delta _{11}=0.3515 &{} \delta _{12}=0.2463 &{} \delta _{13}=0.4022 \\ \delta _{21}=0.3355 &{} \delta _{22}=0.3344 &{} \delta _{23}=0.3301 &{} \delta _{21}=0.3525 &{} \delta _{22}=0.2510 &{} \delta _{23}=0.3964 \\ \delta _{31}=0.3317 &{} \delta _{32}=0.3422 &{} \delta _{33}=0.3261 &{} \delta _{31}=0.3496 &{} \delta _{32}=0.2576 &{} \delta _{33}=0.3928 \\ \delta _{41}=0.3266 &{} \delta _{42}=0.3392 &{} \delta _{43}=0.3342 &{} \delta _{41}=0.3435 &{} \delta _{42}=0.2548 &{} \delta _{43}=0.4017 \\ \end{array} \end{aligned}$$

**Table 9:** Decision matrix

	$C_1$	$C_2$	$C_3$
$A_1$	$<(3,0.4), (0.4,0.2,0.3)>$	$<(7, 0.6),(0.4,0.1,0.2)>$	$<(5, 0.4),(0.7,0.2,0.4)>$
$A_2$	$<(4,0.2), (0.6,0.1,0.2)>$	$<(8, 0.4),(0.6,0.1,0.2)>$	$<(6, 0.7),(0.3,0.5,0.8)>$
$A_3$	$<(3.5,0.3), (0.3,0.2,0.3)>$	$<(6, 0.2),(0.5,0.2,0.3)>$	$<(5.5, 0.6),(0.4,0.2,0.7)>$
$A_4$	$<(5,0.5), (0.7,0.1,0.2)>$	$<(7, 0.5),(0.6,0.1,0.1)>$	$<(4.5, 0.5),(0.6,0.3,0.7)>$

**Table 10:** Normalized decision matrix

	$C_1$	$C_2$	$C_3$
$A_1$	$<(0.6,0.1067), (0.4,0.2,0.3)>$	$<(0.875, 0.0875),(0.4,0.1,0.2)>$	$<(0.9, 0.0475),(0.4,0.2,0.7)>$
$A_2$	$<(0.8,0.02), (0.6,0.1,0.2)>$	$<(1, 0.0333),(0.6,0.1,0.2)>$	$<(0.75, 0.1167),(0.8,0.5,0.3)>$
$A_3$	$<(0.7,0.0514), (0.3,0.2,0.3)>$	$<(0.75, 0.0111),(0.5,0.2,0.3)>$	$<(0.818, 0.0935),(0.7,0.2,0.4)>$
$A_4$	$<(1,0.1), (0.7,0.1,0.2)>$	$<(0.875, 0.0595),(0.6,0.1,0.1)>$	$<(1, 0.0794),(0.7,0.3,0.6)>$

**Table 11:** Aggregation results

Weighted	Non-weighted
$<(0.8164,0.0860),(0.4991,0.1482,0.4082)>$	$<(0.8291,0.0874),(0.4890,0.1808,0.4103)>$
$<(0.8682,0.0699),(0.7240,0.2475,0.2128)>$	$<(0.8801,0.0709),(0.7068,0.2467,0.2499)>$
$<(0.8003,0.0547),(0.5643,0.1883,0.2992)>$	$<(0.8094,0.0553),(0.5519,0.2333,0.3307)>$
$<(0.9547,0.0691),(0.7297,0.1534,0.3129)>$	$<(0.9671,0.0700),(0.7141,0.1748,0.3047)>$

**Table 12:** The effect of the R parameter on the SPFPMM results (Investment company)

Parameter	SNoFPMM		SNoWFPMM	
	Score	Rank	Score	Rank
(1, 0, 0)	$S(A_1)=0.9888$	$A_4 \succ A_2 \succ A_3 \succ A_1$	$S(A_1)=0.9663$	$A_4 \succ A_2 \succ A_3 \succ A_1$
	$S(A_2)=1.3398$		$S(A_2)=1.3377$	
	$S(A_3)=1.1190$		$S(A_3)=1.1286$	
	$S(A_4)=1.3520$		$S(A_4)=1.3427$	
(1, 1, 0)	$S(A_1)=0.9579$	$A_4 \succ A_2 \succ A_3 \succ A_1$	$S(A_1)=0.9314$	$A_4 \succ A_2 \succ A_3 \succ A_1$
	$S(A_2)=1.2710$		$S(A_2)=1.2330$	
	$S(A_3)=1.0644$		$S(A_3)=1.0317$	
	$S(A_4)=1.3026$		$S(A_4)=1.2762$	
(1, 1, 1)	$S(A_1)=0.9313$	$A_4 \succ A_2 \succ A_3 \succ A_1$	$S(A_1)=0.9139$	$A_4 \succ A_2 \succ A_3 \succ A_1$
	$S(A_2)=1.2378$		$S(A_2)=1.1935$	
	$S(A_3)=1.0400$		$S(A_3)=0.9926$	
	$S(A_4)=1.2600$		$S(A_4)=1.2303$	

*Step 5.* There are aggregation results in Table 11. Weighted and non-weighted respectively.

*Step 6.* Score values are calculated.

*Step 7.* Ranking alternatives

Non-weighted: $$A_4=1.3975 \succ A_2=1.3676 \succ A_3=1.1566 \succ A_1=1.0502$$.

Weighted: $$A_4=1.3690 \succ A_2=1.3267 \succ A_3=1.1127 \succ A_1=1.0311$$.

If Tables 12 and 13 are taken into consideration, it is seen that the ideal company for investment is the arms company. On the other hand, it can be seen that the food company is ideal for investment if there is only one criterion relationship in Table 13. Considering the Table 12, considering the interactions of the criteria, the ideal alternative, in any case, is obtained as $A_4$. Also, even if the weights are included, this ranking does not change. On the other hand, in Table 13, there are dual structures of the same operators. Similarly, in Dual structures, the ideal alternative does not change. In both tables (Tables 12, 13), the score values of $A_2$ and $A_4$ are close to each other. however, it can be seen that the difference between the $A_2$ and $A_4$ alternatives increases as the interaction between the criteria increases. This situation shows the importance of the effect of all criteria.

When the ideal investment company selection problem is considered weighted and unweighted, the ideal alternative is $A_4$. The choice of the ideal investment company can be changed with the change of the parameter vector of the Muirhead Mean operator because the parameter vector examines the relationship between any number of criteria. Since there are three criteria in this problem, the size of the parameter vector is three. The used weights of the power aggregation operator and the weights obtained on the decision matrix provide a more stable solution to the real-world problem.

**Table 13:** The effect of the R parameter on the SPFPDMM results (Investment company)

Parameter	SNoFPDMM		SNoFWDPMM	
	Score	Rank	Score	Rank
(1, 0, 0)	$S(A_1)=0.8874$	$A_2 \succ A_4 \succ A_3 \succ A_1$	$S(A_1)=0.8635$	$A_2 \succ A_4 \succ A_3 \succ A_1$
	$S(A_2)=1.2372$		$S(A_2)=1.2301$	
	$S(A_3)=1.0526$		$S(A_3)=1.0559$	
	$S(A_4)=1.2223$		$S(A_4)=1.2071$	
(1, 1, 0)	$S(A_1)=0.9869$	$A_4 \succ A_2 \succ A_3 \succ A_1$	$S(A_1)=1.0011$	$A_4 \succ A_2 \succ A_3 \succ A_1$
	$S(A_2)=1.3130$		$S(A_2)=1.3229$	
	$S(A_3)=1.1020$		$S(A_3)=1.1188$	
	$S(A_4)=1.3299$		$S(A_4)=1.3435$	
(1, 1, 1)	$S(A_1)=1.0254$	$A_4 \succ A_2 \succ A_3 \succ A_1$	$S(A_1)=1.0592$	$A_4 \succ A_2 \succ A_3 \succ A_1$
	$S(A_2)=1.3320$		$S(A_2)=1.3451$	
	$S(A_3)=1.1153$		$S(A_3)=1.1358$	
	$S(A_4)=1.3661$		$S(A_4)=1.3848$

**Table 14:** Rank comparison with other methods

Methods	Ranking
Correlation coefficients of normal neutrosophic sets [65]	$A_4 \succ A_2 \succ A_1 \succ A_3$
Normal neutrosophic Bonferroni mean operators [30]	$A_4 \succ A_2 \succ A_3 \succ A_1$
Normal neutrosophic generalized weighted power averaging operator [33]	$A_4 \succ A_2 \succ A_3 \succ A_1$
Normal neutrosophic generalized prioritized aggregation operators [43]	$A_4 \succ A_2 \succ A_3 \succ A_1$
SNoFPMM and SNoFWPMM	$A_4 \succ A_2 \succ A_3 \succ A_1$
SNoFPDMM and SNoFWPDMM	$A_4 \succ A_2 \succ A_3 \succ A_1$

Considering Table 14, the proposed aggregation operators are in the same ranking as other methods. Moreover, the normal power Muirhead Mean operator includes normal Bonferroni mean and power averaging operators. The most ideal company for investment is chosen as the arms company.

**Table 15:** Validity test results for criteria 2 and 3. (COVID and Investment datasets respectively.)

Methods	Score and Ranking
SNoFPMM	$S(A_1)$ = 0.5289, $S(A_2)$ = 0.7983, $S(A_3)$ = 0.8135, $S(A_4)$ = 0.7941, $S(A_6)=$ 0.6027,$S(A_{5_n})$ = 0.5224
	$$A_3 \succ A_2 \succ A_4 \succ A_6 \succ A_1 \succ A_5$$
SNoWFPMM	$S(A_1)$ = 0.5107, $S(A_2)$ = 0.7519, $S(A_3)$ = 0.7739, $S(A_4)$ = 0.7884, $S(A_6)=$ 0.5798,$S(A_{5_n})$ = 0.4940
	$$A_4 \succ A_3 \succ A_2 \succ A_6 \succ A_1 \succ A_5$$
SNoFPDMM	$S(A_1)$ = 0.6395, $S(A_2)$ = 0.8739, $S(A_3)$ = 0.9104, $S(A_4)$ = 1.1023, $S(A_6)=$ 0.7548,$S(A_{5_n})$ = 0.5970
	$$A_4 \succ A_3 \succ A_2 \succ A_6 \succ A_1 \succ A_5$$
SNoFWDPMM	$S(A_1)$ = 0.6844, $S(A_2)$ = 0.8994, $S(A_3)$ = 0.9270, $S(A_4)$ = 1.1351, $S(A_6)=$ 0.7800,$S(A_{5_n})$ = 0.6252
	$$A_4 \succ A_3 \succ A_2 \succ A_6 \succ A_1 \succ A_5$$
SNoFPMM	$S(A_1)=$ 0.9414, $S(A_2)=$ 1.2378, $S(A_3)=$ 1.0400, $S(A_4)=$ 1.2600
	$$A_4 \succ A_2 \succ A_3 \succ A_1 $$
SNoWFPMM	$S(A_1)=$ 0.9284, $S(A_2)=$ 1.1935, $S(A_3)=$ 0.9926, $S(A_4)=$ 1.2303
	$$A_4 \succ A_2 \succ A_3 \succ A_1 $$
SNoFPDMM	$S(A_1)=$ 1.0202, $S(A_2)=$ 1.3320, $S(A_3)=$ 1.1153, $S(A_4)=$ 1.3661
	$$A_4 \succ A_2 \succ A_3 \succ A_1 $$
SNoFWDPMM	$S(A_1)=$ 1.0482, $S(A_2)=$ 1.3451, $S(A_3)=$ 1.1358, $S(A_4)=$ 1.3848
	$$A_4 \succ A_2 \succ A_3 \succ A_1 $$

## Validity test for irregularities

There are test criteria created to test whether the proposed method is valid in MCDM [53]. We will use these criteria to test the robustness of the methods we have created.

*Criterion 1:* “The best alternative should not change when a non-optimal alternative is altered with an alternative that has lower score values.”

*Criterion 2:* “Transitive feature should be provided”

**Table 16:** Validity test results for criterion 1 (COVID and Investment datasets respectively)

Subset	Non-weighted WTSPFPMM	Weighted WTSPFPMM	Non-weighted WTSPFPDMM	Weighted WTSPFPDMM
$A_2, A_3, A_4, A_5, A_6$	$A_3 \succ A_2 \succ A_4 \succ A_6 \succ A_5$	$A_4 \succ A_3 \succ A_2 \succ A_6 \succ A_5$	$A_4 \succ A_3 \succ A_2 \succ A_6 \succ A_5$	$A_4 \succ A_3 \succ A_2 \succ A_6 \succ A_5$
$A_1, A_3, A_4, A_5, A_6$	$A_3 \succ A_4 \succ A_6 \succ A_1 \succ A_5$	$A_4 \succ A_3 \succ A_6 \succ A_1 \succ A_5$	$A_4 \succ A_3 \succ A_6 \succ A_1 \succ A_5$	$A_4 \succ A_3 \succ A_6 \succ A_1 \succ A_5$
$A_1, A_2, A_4, A_5, A_6$	$A_2 \succ A_4 \succ A_6 \succ A_1 \succ A_5$	$A_4 \succ A_2 \succ A_6 \succ A_1 \succ A_5$	$A_2 \succ A_4 \succ A_6 \succ A_1 \succ A_5$	$A_2 \succ A_4 \succ A_6 \succ A_1 \succ A_5$
$A_1, A_2, A_3, A_5, A_6$	$A_3 \succ A_2 \succ A_6 \succ A_1 \succ A_5$	$A_3 \succ A_2 \succ A_6 \succ A_1 \succ A_5$	$A_3 \succ A_2 \succ A_6 \succ A_1 \succ A_5$	$A_3 \succ A_2 \succ A_6 \succ A_1 \succ A_5$
$A_1, A_2, A_3, A_4, A_6$	$A_3 \succ A_2 \succ A_4 \succ A_6 \succ A_1$	$A_4 \succ A_3 \succ A_2 \succ A_6 \succ A_1$	$A_4 \succ A_3 \succ A_2 \succ A_6 \succ A_1$	$A_4 \succ A_3 \succ A_2 \succ A_6 \succ A_1$
$A_1, A_2, A_3, A_4, A_5$	$A_3 \succ A_2 \succ A_4 \succ A_1 \succ A_5$	$A_4 \succ A_3 \succ A_2 \succ A_1 \succ A_5$	$A_4 \succ A_3 \succ A_2 \succ A_1 \succ A_5$	$A_4 \succ A_3 \succ A_2 \succ A_1 \succ A_5$
$A_2, A_3, A_4$	$A_4 \succ A_2 \succ A_3$	$A_4 \succ A_2 \succ A_3$	$A_4 \succ A_2 \succ A_3$	$A_4 \succ A_2 \succ A_3$
$A_1, A_3, A_4$	$A_4 \succ A_3 \succ A_1$	$A_4 \succ A_3 \succ A_1$	$A_4 \succ A_3 \succ A_1$	$A_4 \succ A_3 \succ A_1$
$A_1, A_2, A_4$	$A_4 \succ A_2 \succ A_1$	$A_4 \succ A_2 \succ A_1$	$A_4 \succ A_2 \succ A_1$	$A_4 \succ A_2 \succ A_1$
$A_1, A_2, A_3$	$A_2 \succ A_3 \succ A_1$	$A_2 \succ A_3 \succ A_1$	$A_2 \succ A_3 \succ A_1$	$A_2 \succ A_3 \succ A_1$

*Criterion 3:* “When the problem is broken down into small parts, the ordering of the sub-problems should coincide with the ordering of the problem.”

The proposed methods show us that alternatives $A_5$ for the COVID dataset and $A_1$ for the Investment dataset are not ideal alternatives because they are last in the ranking results. Therefore, we can choose worse alternatives than these and repeat our experiments with new alternatives. Criterion 1 is met if the newly obtained results support the previous results. The R value to be used in validity tests is the [1,$\ldots$,1] value where all criteria are included in the calculation.

Let $$A_{5_n}=$$ $$<(115,11),(0.39,0.58,0.64)>
<(40,4.3),(0.34,0.66,0.43)>
<(85,8.2),(0.45,0.68,0.34)>
<(6.5,0.71),(0.23,0.61,0.61)>$$ and $$A_{1_n}=$$ $$<(2,0.4),(0.4, 0.2, 0.3)>
<(6, 0.6),(0.4, 0.1, 0.2)>
<(4.6, 0.45), (0.7, 0.2, 0.4)>$$ be the new alternatives instead of $$A_5$$ in the COVID dataset, and $$A_1$$ in the Investment dataset.

When the new results are examined, $A_{5_n}$ and $A_{1_n}$ are again in the last place as seen in Table 15. As a result, criterion 1 is provided. When the data set is divided into sub-samples and then ranked according to the suggested methods, we obtain the result in Table 16. The results are equivalent to the experiments performed on the entire dataset. Therefore, it seems that criteria 2 and 3 have been met.

## Conclusions

Aggregation operators directly affect the decision-making process. Therefore, aggregation operators are frequently used in solving MADM problems. Generally, in MADM problems, people’s personal decisions are evaluated. This situation requires the examination of uncertainty and incomplete data in the decision matrix created by an expert or a decision-maker. Therefore, using aggregation operators and generalized fuzzy sets for solving MADM problems provides sensible and consistent solutions. In the proposed methods, superior aspects of both aggregation operators and generalized fuzzy sets are analyzed. The novelty in the article is that the power Muirhead Mean operator is handled in a spherical normal fuzzy environment.

The advantages of the SNoFPMM and SNoFWPMM aggregation operators are stated below.

1. With the power aggregation operator, the negative effect of extremely high or low values is reduced.
2. Spherical fuzzy sets provide decision-makers with a wide range of evaluations. Independent hesitant degree also handles uncertain data more consistently.
3. Normal fuzzy sets are compatible with their variance and mean values for real-life data. SNoFPMM operators have also proven to be a spherical fuzzy set.
4. The MM operator allows the interrelationship between all characters to be taken into account. It also includes the MM operator, Maclaurin Symmetric Mean, and Bonferroni mean operators.
5. Finally, the SNoFPMM and SNoFWPMM operators apply to the problems of choosing the ideal mask for protection from the COVID-19 outbreak and investment company selection.

The SNoFPMM and SNoFWPMM aggregation operators produced similar ranking results, with and without weight. Therefore, these operators can be evaluated independently of their weight. On the other hand, a detailed parameter analysis was made with the parameter vector of the MM operator. Similar ranking results were obtained when the proposed methods were compared with other studies. Besides, SNoFPMM and SNoFWPMM have many superior aspects than others. However, this study has some limitations. The examples in this article consist of up to four criteria. Since the article uses the power Muirhead Mean aggregation operator, the running time will increase as the number of criteria increases. Because ${n\atopwithdelims ()2}$ different cases must be calculated for n different criteria. Also, since Muirhead Mean calculates all permutations, the number of criteria increases the complexity of the problem. Normal fuzzy sets can be used in neutrosophic [27], interval neutrosophic sets [35]. There are also various studies on real-world problems with neutrosophic and hypersoft sets [19–21, 42]. Normal Muirhead Mean operators can be applied to hypersoft sets. The proposed method can be combined with the TOPSIS method in an interval-valued intuitionistic fuzzy set environment [23] for future studies. On the other hand, the SNoFPMM operator can be applied to T-norm operators like Dombi [7], Hamacher [12], which have flexible parameters. In addition, these operators can also be expanded to the bipolar environment [1].
